# A Novel Gliotransmitter, L-β-Aminoisobutyric Acid, Contributes to Pathophysiology of Clinical Efficacies and Adverse Reactions of Clozapine

**DOI:** 10.3390/biom13091288

**Published:** 2023-08-23

**Authors:** Kouji Fukuyama, Eishi Motomura, Motohiro Okada

**Affiliations:** Department of Neuropsychiatry, Division of Neuroscience, Graduate School of Medicine, Mie University, Tsu 514-8507, Japan; k-fukuyama@clin.medic.mie-u.ac.jp (K.F.); motomura@clin.medic.mie-u.ac.jp (E.M.)

**Keywords:** clozapine L-β-aminoisobutyric acid, treatment-resistant schizophrenia, metabolic complication, thalamocortical pathway

## Abstract

Clozapine is listed as one of the most effective antipsychotics and has been approved for treating treatment-resistant schizophrenia (TRS); however, several type A and B adverse reactions, including weight gain, metabolic complications, cardiotoxicity, convulsions, and discontinuation syndromes, exist. The critical mechanisms of clinical efficacy for schizophrenia, TRS, and adverse reactions of clozapine have not been elucidated. Recently, the GABA isomer L-β-aminoisobutyric acid (L-BAIBA), a protective myokine in the peripheral organs, was identified as a candidate novel transmission modulator in the central nervous system (CNS). L-BAIBA activates adenosine monophosphate-activated protein kinase (AMPK) signalling in both the peripheral organs and CNS. Activated AMPK signalling in peripheral organs is an established major target for treating insulin-resistant diabetes, whereas activated AMPK signalling in the hypothalamus contributes to the pathophysiology of weight gain and metabolic disturbances. Clozapine increases L-BAIBA synthesis in the hypothalamus. In addition, the various functions of L-BAIBA in the CNS have recently been elucidated, including as an activator of GABA-B and group-III metabotropic glutamate (III-mGlu) receptors. Considering the expressions of GABA-B and III-mGlu receptors (localised in the presynaptic regions), the activation of GABA-B and III-mGlu receptors can explain the distinct therapeutic advantages of clozapine in schizophrenia or TRS associated with N-methyl-D-aspartate (NMDA) receptor disturbance compared with other atypical antipsychotics via the inhibition of the persistent tonic hyperactivation of thalamocortical glutamatergic transmission in the prefrontal cortex. L-BAIBA has also been identified as a gliotransmitter, and a detailed exploration of the function of L-BAIBA in tripartite synaptic transmission can further elucidate the pathophysiology of effectiveness for treating TRS and/or specific adverse reactions of clozapine.

## 1. Introduction

Traditionally, more than 30% of patients with schizophrenia spectrum are considered to suffer from treatment-resistant schizophrenia (TRS) [1,2,3]. Clozapine is evaluated as the most effective antipsychotic agent for TRS since 30–60% of patients with TRS respond to clozapine medication [4,5,6]. Therefore, clozapine is currently the only approved antipsychotic for TRS treatment [7]. In fact, several guidelines recommend initiating treatment with clozapine for patients with TRS [8,9,10]. Furthermore, systematic reviews and meta-analyses have demonstrated that clozapine is associated with lower hospitalisation rates, lower overall discontinuation rates, and better overall symptom outcomes compared with other atypical antipsychotics [11,12,13].

All antipsychotics approved for the treatment of schizophrenia are antagonists of the dopamine D2 receptor at therapeutically relevant concentrations [14,15]. The introduction of clozapine in the 1970s marked a significant turning point in the pharmacotherapy of schizophrenia. As an alternative, clozapine minimised the risk of extrapyramidal symptoms, such as antipsychotic-induced parkinsonism and tardive dyskinesia, while demonstrating excellent efficacy for both positive and negative symptoms of schizophrenia [16,17]. Based on these clinical advantages of clozapine, receptor-binding profile screenings have contributed to the development of several second-generation antipsychotics (atypical antipsychotics) that share pharmacological characteristics distinct from the preceding first-generation antipsychotics (typical antipsychotics) (Table 1) [16,18]. It is well known that olanzapine has a similar receptor-binding profile to clozapine, except for the 5-HT7 receptor [19] (Table 1); however, the specific effectiveness of clozapine for treating TRS suggests the pathophysiology of clozapine may involve molecules other than monoamine receptors.

Most atypical antipsychotics had been developed by exploring molecules that have similar receptor-binding profiles to clozapine that are distinct from the preceding typical antipsychotics, such as having a relatively lower binding affinity to the dopamine D2 receptor and higher affinity to serotonin 5-HT2A receptors [20]. Therefore, the pathophysiological hypothesis proposed to distinguish between typical and atypical antipsychotics, having a relatively low affinity to the D2 receptor and relatively high affinity to the 5-HT2A receptor, cannot account for the distinct therapeutic advantages of clozapine against other atypical antipsychotics.

**Table 1:** Receptor-binding profiles of antipsychotics.

Receptor	CLZ	LUR	APZ	Brex	OLZ	QTP	RIS	ZTP	HPD
5-HT1A	124	6.8	5.6	0.12	>1000	432	423	471	>1000
5-HT2A	5.4	2.0	8.7	0.47	2.3	100	0.2	2.7	53
5-HT2C	9.4	415	76	63	14	>1000	12	2.6	>1000
5-HT7	18.0	0.5	10.3	3.7	365	307	6.6	12.0	377
H1	1.13	>1000	27.6	19	1.2	11	20.1	3.21	>1000
D1	266	262	>1000	160	100	712	244	71.0	80
D2	157	1.7	3.3	0.3	52.3	245	3.6	25.0	0.7
References	[21,22]	[23]	[24,25]	[26]	[27,28]	[29]	[25,30]	[31]	[32,33]

Clozapine (CLZ), lurasidone (LUR), aripiprazole (APZ), brexpiprazole (Brex), olanzapine (OLZ), quetiapine (QTP), risperidone (RIS), zotepine (ZTP), and haloperidol (HPD) against serotonin (5-HT) type 1A (5-HT1A), type 2A (5-HT2A), type 2C (5-HT2C), and type 7 (5-HT7) receptors, histamine H1 (H1) receptor, and dopamine receptors type 1 (D1) and 2 (D2). Data are equilibrium constant (Ki) values (nM).

Despite the clinical advantages of clozapine’s effectiveness for TRS, clozapine is also associated with numerous specific/serious adverse reactions, such as type B reactions (agranulocytosis, eosinophilia and haematological malignancies, myocarditis, cardiomyopathy, and convulsions) and type A reactions (weight gain and metabolic disturbance) [34,35,36,37,38]. Occasionally, psychiatrists must promptly discontinue clozapine or switch to other antipsychotics due to these lethal type B adverse reactions. However, prompt discontinuation often leads to clozapine discontinuation symptoms, including clozapine-discontinuation-induced worsening of psychosis and catatonia [38,39,40]. These distinct clinical advantages and disadvantages of clozapine compared with conventional atypical antipsychotics suggest that clozapine likely has different mechanisms of action compared with other atypical antipsychotics.

Tripartite synaptic transmission involving molecules other than monoamine receptors has recently been speculated to play important roles in the pharmacological mechanisms of clozapine’s therapeutic effects and adverse reactions, which is distinct from other atypical antipsychotics [38,41,42,43,44,45,46,47]. In these pharmacodynamic researches, it has been identified that L-β-aminoisobutyric acid (L-BAIBA) plays an important role in the pathophysiology underlying the mechanisms of clozapine’s clinical efficacy for the treatment of schizophrenia and TRS as well as in adverse reactions, such as weight gain and metabolic complications [46,47]. In other words, L-BAIBA is a candidate target molecule that can rationally explain the mechanisms of clozapine’s therapeutic effects and adverse reactions, which have not been elucidated so far, via modulating various signalling pathways. In this review, we outline the characteristics of the therapeutic effects and adverse reactions of clozapine and then propose a compelling pathophysiological hypothesis that L-BAIBA is involved in the underlying mechanism of clozapine, which has not been elucidated.

## 2. Clozapine-Induced Metabolic Complications

Weight gain is the most prevalent adverse reaction of atypical antipsychotic medications. Weight gain induced by atypical antipsychotics usually occurs during the early stages of antipsychotic treatment (within the first year), with an increase of 7% over baseline weight observed in approximately two-thirds of antipsychotic-treated patients [48,49]. Diabetes treatment in patients treated with clozapine is manageable by following current diabetes treatment guidelines [50,51]. Thus, a history of diabetes in TRS patients does not constitute a contraindication to clozapine medication [52]. Among pharmacological interventions, metformin has an excellent safety profile and is the most effective for weight gain stabilisation [53,54,55]. Topiramate has also been demonstrated to be as effective as metformin in suppressive effects on clozapine-induced weight gain [56,57]. Glucagon-like peptide-1 (GLP1) receptor agonists have been recently shown to effectively mitigate clozapine-induced metabolic disturbances [58]. However, weight gain induced by antipsychotics other than clozapine, including olanzapine and quetiapine, reaches a plateau within the therapeutic dose range, whereas the unique features of weight gain with clozapine indicate a linear dose-dependent manner ranging from therapeutic to supratherapeutic doses [59]. This specific linear dose-dependent weight gain induced by clozapine indicates that different mechanisms might underlie the weight gain induced by other antipsychotics.

**Figure 1:**
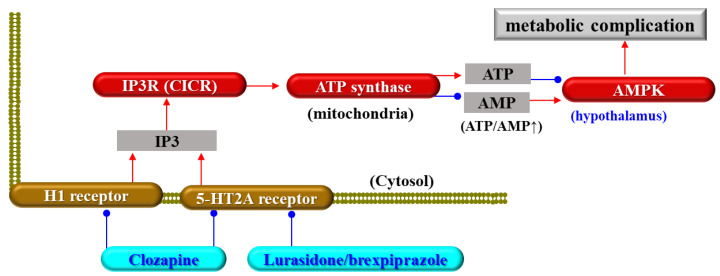
Schematic presentation of hypothalamic signalling associated with traditional hypothesis regarding the mechanisms of antipsychotic-induced metabolic complications and weight gain. Red and blue arrows indicate activation and inhibition, respectively. Abbreviations: H1 receptor—histamine H1 receptor, 5-HT2A receptor—serotonin 5-HT2A receptor, IP3—of inositol trisphosphate, CICR—Ca^2+^-induced Ca^2+^-releasing system, ATP—adenosine triphosphate, AMP—adenosine monophosphate, and AMPK—AMP-activated protein kinase.

Atypical antipsychotic-induced metabolic complications have been considered to be related to the inhibition of the histamine H1 and serotonin 5-HT2A receptors, which leads to the disturbance of energy regulation systems in the hypothalamus [60,61]. The inhibition of the H1 and 5-HT2A receptors suppresses the synthesis of inositol trisphosphate (IP3), which activates the calcium-induced calcium-releasing system (CICR) via the enhancement of the IP3 receptor (Figure 1) [62,63]. The elevation in intracellular calcium ion levels activates adenosine triphosphate (ATP) synthase, leading to an increase in ATP and/or a decrease in adenosine monophosphate (AMP) levels (Figure 1) [61,63,64]. Therefore, CICR suppression induced by H1 and 5-HT2A receptor inhibition secondarily increases intracellular AMP levels, leading to the activation of adenosine monophosphate (AMP)-activated protein kinase (AMPK) (Figure 1) [46,60,61,64]. This hypothesis has been supported by the clinical findings on high-affinity H1 and 5-HT2A receptor antagonistic antipsychotics, including zotepine, quetiapine, olanzapine, and clozapine listed as being high-risk for metabolic complications [59]. However, the activation of AMPK in the peripheral organs is one of the major therapeutic targets for insulin-resistant diabetes [55,65,66], whereas the activation of AMPK signalling in the hypothalamus increases feeding and reduces energy expenditure in the body [66].

Chronic administration of therapeutically relevant doses of clozapine, quetiapine, brexpiprazole, and lurasidone decreased IP3 synthesis, and increased AMP levels in the rat hypothalamus [46,47,67]. However, contrary to expectations, AMPK signallings were activated and unaffected by high-risk (clozapine and quetiapine) and low-risk (brexpiprazole and lurasidone) antipsychotics for weight gain, respectively [44,45,46,47,68]. Both clozapine and quetiapine are high-affinity antagonists of the histamine H1 receptor and the 5-HT2A receptor, whereas brexpiprazole and lurasidone are high-affinity 5-HT2A receptors but have low binding affinity to the H1 receptor [60,69]. Therefore, enhanced intra-hypothalamic AMPK signalling plays fundamental roles in antipsychotic-induced metabolic complications and weight gain, but decreasing IP3 with increasing AMP levels via inhibition of H1 and/or 5-HT2A receptors alone cannot explain the pathophysiology of antipsychotic-induced weight gain. Similar to clozapine, an H1 and 5-HT2A high-affinity atypical antipsychotic agent, olanzapine, which was established to also be a high-risk antipsychotic for weight gain, decreased IP3 synthesis [70,71]; however, olanzapine has been reported to enhance [61,72,73] and suppress [74,75] hypothalamic AMPK signalling with contradictory results. A recent study by Ferreira et al., which set the plasma concentration of olanzapine ranging from the maximum therapeutic concentration to the supratherapeutic level, demonstrated that olanzapine suppressed the hypothalamic AMPK signalling [75]. Considering that, clinically, olanzapine (lower than 10 mg/day) dose-dependently increased body weight, but above 10 mg/day, the weight gain induced by olanzapine displayed plateaus [59]; olanzapine has a dose-dependent biphasic effect on hypothalamic AMPK signalling, with activation by low-dose and suppression by high-dose. Further studies need to clarify these our hypothesis and the detailed mechanisms of dose-dependent biphasic action of olanzapine on AMPK signalling in the hypothalamus.

## 3. Clozapine and TRS

### 3.1. Efficacy of Clozapine in TRS

TRS is internationally defined by the Treatment Response and Resistance in Psychosis (TRRIP) Working Group and includes the following aspects: the presence of persistent symptoms—including positive and negative symptoms, and cognitive impairment—over at least 12 weeks of at least moderate severity caused by moderate levels of functional impairments [76]. Symptom classifications and thresholds should be based on standardised and validated clinical rating scales. Insufficient response to medication with at least two different antipsychotic medications, with a minimum treatment duration of twelve weeks (six weeks for each antipsychotic agent). This corresponds to a minimum dose equivalent to 600 mg per day of chlorpromazine. Confirmation of adequate treatment adherence is defined as the patient having taken at least 80% of the prescribed dose. To achieve this, at least two methods should be employed, including counting tablets, patient and caregiver reports, and review of medical records and documentation. Additionally, plasma drug concentrations should be monitored at least once for each antipsychotic agent [76,77].

Incontrovertible evidence supports the superior efficacy of clozapine compared with other atypical antipsychotics in improving positive symptoms and global psychopathology in TRS [5,13,78,79]. Considering the lack of evidence to support using polypharmacy of antipsychotics other than clozapine that is as effective as clozapine, the efficacy of clozapine in TRS is evaluated as being more robust [80]. Furthermore, patients treated with clozapine have also shown improvements in treatment adherence, resulting in decreased rehospitalisation rates [80,81].

### 3.2. Candidate Pathophysiology of TRS

Some research groups have emphasised the importance of distinguishing between primary and secondary TRS: primary TRS already presents with antipsychotic-resistant clinical features at the onset of the schizophrenia spectrum, whereas secondary TRS develops at later stages of the schizophrenia spectrum after an initial adequate response to antipsychotics [82,83,84]. Dopaminergic supersensitivity induced by consecutive exposure to antipsychotics has been speculated as a candidate mechanism of secondary TRS [85]. Persistent exposure to antipsychotics upregulates postsynaptic D2 receptors, leading to further psychotic exacerbation [85]. The estimated overall response rate to antipsychotic medications ranges from 40% to 60% [86,87]. The response rate to antipsychotic medication in antipsychotic-naïve patients is estimated to be approximately 75%; however, the response rate in a second trial using antipsychotic medications other than clozapine was considerably lower, ranging from 20% to 45% [88,89]. Response rates to clozapine have been reported to be maximally up to 80% when treatment is initiated within the first 2–3 years after resistance is established [87,88,90]. With subsequent initiation of clozapine medication, the response rate might be as low as 30% [87]. The efficacy of clozapine against TRS is significant compared with other antipsychotics but decreases depending on the duration of antipsychotic exposure, which is similar to other antipsychotics. These clinical findings regarding duration-dependent resistance at least partially support the dopaminergic supersensitivity hypothesis [85].

The specific features of clozapine, such as low affinity and rapid dissociation from D2 receptors, are considered to be candidate mechanisms via which clozapine-induced D2 receptor supersensitivity is less than that of other antipsychotics [20,38,69,91,92]. However, several line studies have demonstrated that the dissociation rate of clozapine from D2 receptors is not significantly faster compared with the rates of other antipsychotics, such as quetiapine, amisulpride, remoxipride, and sulpiride [20,93,94,95]. These pharmacodynamic findings suggest that the efficacy of clozapine in secondary TRS cannot be solely explained by either its low affinity or rapid dissociation from D2 receptors, even if the pathophysiology of TRS involves D2 receptor supersensitivity.

### 3.3. Candidate Targets of Clozapine Other Than Monoamine Receptors

Although schizophrenia is commonly speculated to be a pathophysiologically contiguous spectrum between treatment-responsive schizophrenia and TRS, several findings suggest that TRS might be a subtype with extreme characteristics from the perspective of neurodevelopmental disorders [96,97]. In other words, there are possibly two subtypes of pathophysiology of TRS, one being secondary treatment resistance due to long-term exposure to antipsychotic drugs, and the other already developing as TRS during the onset period. Approximately 70–80% of patients with TRS have been reported to present antipsychotic-resistant clinical features from the first episode [96,97]. Furthermore, predictors of antipsychotic resistance in schizophrenia are similar to the clinical features of ‘neurodevelopmental’ schizophrenia, such as being male, being of a younger age at onset, poor premorbid adjustment, and a longer duration of untreated illness [98,99]. So far, various studies have revealed impairments in cognitive components, such as sensorimotor function, attention, working memory, visuospatial processing, verbal intelligence, and memory in TRS patients compared with treatment-responsive schizophrenia [100,101,102,103]. These cognitive impairments are more suggestive of impaired function of glutamate transmission (via thalamocortical pathways) than monoamine transmission (via the mesolimbic and mesocortical systems). These cognitive impairment features of TRS suggest it may be caused by dysfunction of glutamatergic transmission (via thalamocortical pathways) rather than monoaminergic transmission (via mesolimbic and mesocortical pathways) [15,38,41,42,43,44,104].

Quantitative reviews of mRNA and protein expression of N-methyl-D-aspartate glutamate receptor (NMDA-R) in post-mortem studies have demonstrated that both mRNA and protein expression of the NR1 subunit of NMDA-R in the prefrontal cortex decreased in patients with schizophrenia compared with healthy volunteers [105]. mGluR5 (I-mGluR) signalling in the dorsolateral prefrontal cortex decreased, indicating that NMDA-R hypofunctions [106]. In the post-mortem frontal cortex of untreated patients with schizophrenia, downregulation of group II metabotropic glutamate receptors (II-mGluR), such as mGlu2/3, was reported [107]. Conversely, III-mGlu receptor expression in schizophrenia remains unreported, whereas the activation of the III-mGlu receptor suppressed the hyperactivated transmission induced by NMDA-R impairment in wild-type and II-mGluR deficit models [42,108].

In other line studies, both post-mortem and experimental animal model studies also demonstrated that impairment of the GABA-B receptor plays an important role in the pathophysiology of schizophrenia. Decreased GABA-B receptor expression in the hippocampus, prefrontal cortex, inferior temporal cortex, and entorhinal cortex in schizophrenia has been reported [109,110]. Decreased GABA-B receptor expression in the prefrontal cortex and hippocampus of the DBA/2J schizophrenia model compared with C57BL/6J mice was also revealed [111]. Clinically, clozapine is evaluated as the most effective antipsychotic to improve sensorimotor gating dysfunction in patients with schizophrenia [112]. Maladaptive perseveration with strategies that cannot lead to the desired outcome resulting from cognitive and behavioural inflexibility via possible sensorimotor gating dysfunction in the thalamocortical pathway is considered a characteristic feature of schizophrenia [38,60,69,113,114]. Pre-pulse inhibition (PPI) has been established as an endo-phenotype of sensorimotor gating function. Clozapine improved PPI deficits in an experimental animal model, ZFP804A mutant mice, and an NMDA/glutamate receptor (ketamine)-induced model [115,116]. Baclofen has also been indicated to counter PPI disruption of the acoustic startle reflex produced by the blockading of the NMDA-R [111]. Notably, the effects of baclofen on PPI deficit were comparable to those of clozapine but more prominent than those of the typical antipsychotic, haloperidol [111]. These behavioural studies suggest that the impacts of a GABA-B deficit contribute to sensorimotor impairment in schizophrenia.

A recent study using molecular docking calculations for the X-ray crystal structure of the GABA-B receptor suggested that clozapine, like baclofen, might bind to the GABA-B receptor [117]. Both clinical and preclinical studies have suggested that clozapine enhances GABA-B receptor function, and the direct binding of clozapine to the GABA-B receptor has not been demonstrated but, rather, has been denied [59,118,119]. Considering these previous findings, the enhancement of GABA-B receptor function with clozapine may be mediated by an indirect mechanism of clozapine rather than a direct agonist action. Therefore, the hypothesis regarding the stimulatory effects of clozapine on GABA-B receptor function is intriguing for understanding the underlying pathophysiology of the clinical efficacy of clozapine in TRS.

## 4. Impacts of L-BAIBA as the Pharmacodynamic Target of Clozapine

As mentioned above, the exploration of the pathophysiology of schizophrenia has developed over the last fifty years, whereas neither the mechanism of efficacy in TRS nor adverse reactions of clozapine have been clarified [120]. The characteristic mechanisms of clozapine absent in other atypical antipsychotics have not been elucidated [120].

### 4.1. Impacts of L-BAIBA on Metabolic Complications Induced by Clozapine

In 2012, we detected L-BAIBA release in the frontal cortex using microdialysis; however, a detailed release mechanism and function in the brain remain to be clarified [121]. Although the BAIBA enantiomer, a structural GABA isomer, was discovered in human urine in 1951 [122], its function has remained to be elucidated till the 2010s.

In the peripheral organs, BAIBA was rediscovered as a protective myokine that regulates adipose tissue browning, enhances insulin sensitivity, and improves obesity induced by a high-fat diet [123,124,125]. BAIBA increases Akt, AMPK, and insulin receptor substrate signalling and decreases the expression of gluconeogenic enzymes [125]. Activation of AMPK signalling is listed as a major therapeutic target for treating insulin-resistant diabetes [65,66]. Several clinical studies and meta-analyses have reported that antipsychotic-induced weight gain and metabolic complications are meaningfully improved and prevented with the AMPK activator metformin [53,55,126,127,128].

**Figure 2:**
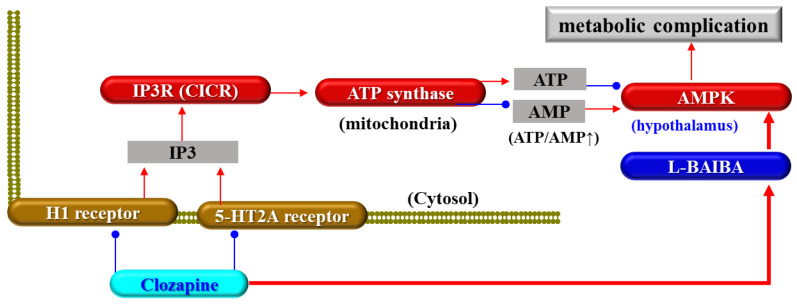
Proposed hypothesis about the mechanism of clozapine-induced metabolic complication of weight gain associated with enhanced L-β-aminoisobutyric acid (L-BAIBA) signalling in the hypothalamus. Red and blue arrows indicate activation and inhibition, respectively.

However, the activation of hypothalamic AMPK signalling may contribute to the pathophysiology of antipsychotic-induced weight gain and metabolic complications [46,60,61,64], since hypothalamic AMPK regulates both sides of the energy balance equation (feeding and energy expenditure) in the body [66]. Many pharmacodynamic studies have revealed that high-risk antipsychotics for weight gain and metabolic complications, such as clozapine, olanzapine, quetiapine, and zotepine activate AMPK signalling, but lower-risk antipsychotics, such as lurasidone and brexpiprazole, decrease and do not affect AMPK signalling in the hypothalamus and other brain regions, respectively [45,46,60,61,68,129,130]. Based on previous clinical and preclinical findings, it is hypothesised that clozapine activates hypothalamic signalling associated with AMPK via enhanced BAIBA signalling. The BAIBA enantiomer activates AMPK signalling in the hypothalamus and astrocytes [46,47,67] (Figure 2). According to our hypothesis, chronic administration of therapeutically relevant doses of clozapine increases the synthesis and release of the BAIBA enantiomer, but neither brexpiprazole nor lurasidone affects BAIBA in the hypothalamus [46,47,67]. This effect of clozapine on the BAIBA enantiomer is primarily on L-BAIBA, whereas the D-BAIBA level in the hypothalamus is lower than the limit of detection [46,47,67]. Therefore, L-BAIBA is a candidate molecule in the brain contributing to the pathophysiology of clozapine-induced weight gain and metabolic complications.

### 4.2. BAIBA Enantiomer Metabolism and Distribution

**Figure 3:**
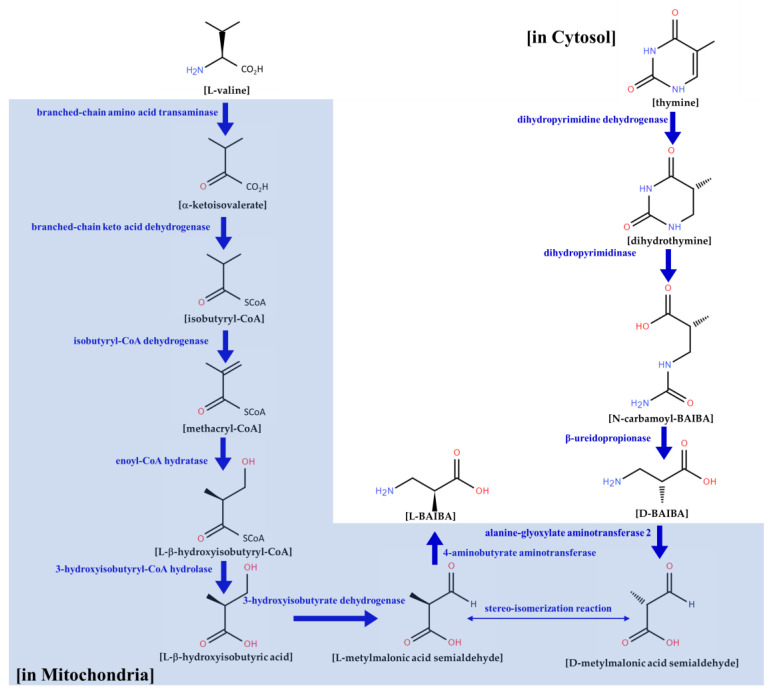
Formation and metabolism of β-aminoisobutyric acid (BAIBA) enantiomer.

There are biologically two BAIBA enantiomers: D-BAIBA (R-BAIBA) and L-BAIBA (S-BAIBA) [131,132]. Although the structures of the BAIBA enantiomers are similar, their metabolic pathways function independently (Figure 3). D-BAIBA is synthesised from thymine and degraded by alanine-glyoxylate aminotransferase-2 [133]. Dihydropyrimidine dehydrogenase (DPYD) generates dihydrothymines from thymine [134]. Dihydropyrimidinase (DPYS) forms N-carbamoyl-β-aminoisobutyric acid (N-carbamoyl-BAIBA) from dihydrothymine. Finally, D-BAIBA is produced by beta-ureidopropionase (UPB1) from N-carbamoyl-BAIBA. This D-BAIBA synthesis process occurs in the cytosol, whereas D-BAIBA is depredated to D-methylmalonic semialdehyde (D-MMS) via glyoxylate aminotransferase 2 (AGXT2). Meanwhile, L-BAIBA is produced via the catabolism of the branched amino acid L-Valine in the mitochondria [135,136,137] (Figure 3). L-Valine is formed via ammonia and the oxidation reaction of methyl malonyl half aldehyde (L-methylmalonylsemialdehyde, L-MMS). L-MMS produces L-BAIBA in a reaction with the mitochondrial enzyme 4-aminobutyrate aminotransferase (ABAT) [138]. It has been reported that the production of L-BAIBA by ABAT is a bidirectional reaction, so the same enzyme can catalyse the conversion of L-BAIBA to L-MMS [135,139]. Furthermore, ABAT depredates GABA [140].

The literature on the distribution of D-BAIBA and L-BAIBA in plasma, urine, and tissues is contradictory. Most studies report that D-BAIBA is the main enantiomer of BAIBA in urine [132,141,142,143,144]. Another study suggested L-BAIBA is the major BAIBA enantiomer in the plasma [132], whereas others have reported that the more prevalent isoform is D-BAIBA [46,47,67,144,145]. Chronic administration of therapeutically relevant doses of clozapine increased L-BAIBA levels but did not affect D-BAIBA levels, resulting in unchanging overall plasma levels of the BAIBA enantiomer [46,47,67].

### 4.3. BAIBA Function in the CNS

Several functions of the BAIBA enantiomer in the CNS have been identified, such as the activation of glycine and GABA-A receptors [146,147]; however, the affinities of the BAIBA enantiomer to these receptors are relatively low, and subsequent functional analysis of the BAIBA enantiomer has not progressed. Clozapine also enhances the III-mGlu receptor and GABA-B receptors and denies direct binding to these receptors [42,108,117,148,149]. Considering that BAIBA is an isomer of GABA and is structurally similar, it is reasonable to predict that it may bind not only to GABA-A receptors but also to GABA-B receptors. The IC50 values of L-BAIBA to the III-mGlu receptor and GABA-B receptors are in approximately micromole orders [47].

The glycine receptor agonistic action of the BAIBA enantiomer can contribute to the interpretation of the pathophysiology of the effectiveness of clozapine in TRS. Various meta-analysis studies have elucidated that candidate NMDA-R modulators, including glycine, D-serine, N-acetyl-cysteine, and sarcosine, have exhibited favourable effects as augmentation therapy for atypical antipsychotics other than clozapine; however, when given to patients intaking clozapine, these modulators cannot improve but rather exacerbate schizophrenia symptoms [150,151,152].

Most neuroscientists have traditionally considered that primary information processing is implemented in the cortex and that the thalamus functions as a communication pathway for sensory input to the cortex [153]. Two signal transformation modes have been typically observed in thalamocortical glutamatergic neurones, bursting and tonic modes. The bursting mode is effective for detecting environmental changes, whereas the tonic mode is suitable for perceptual processing [154]. A well-known hypothesis regarding bottom-up cognition-promoting systems is that the hyperactivation of glutamatergic transmission in thalamocortical pathways plays important roles in several cognitive components, such as sensorimotor gaiting, sensory integration, and executive function [155]. Therefore, the thalamus probably plays an important role in implementing transformations between detection and perception modes [156]. The persistent tonic activation of thalamocortical projections has been observed in several models of schizophrenia, autism spectrum disorders, and attention-deficit/hyperactive disorder [42,60,69,130,157,158,159,160,161,162,163].

**Figure 4:**
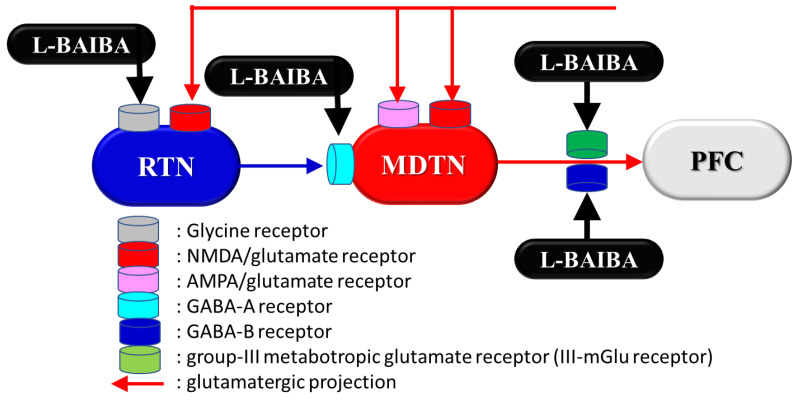
Our proposed hypothesis for the target of action of L-BAIBA on glutamatergic transmission in the thalamocortical pathway. RTN: reticular thalamic nucleus, MDTN: mediodorsal thalamic nucleus, and FPC: prefrontal cortex.

Systemic administration of NMDA-R antagonists drastically enhances thalamic glutamatergic neuronal activities via the inhibition of intra-thalamic GABAergic disinhibition (from the reticular thalamic nucleus to the mediodorsal thalamic nucleus, resulting in the activation of glutamatergic transmission from the thalamus to several cortex regions [42,47,158,164,165,166] (Figure 4)). Several studies have revealed that some atypical antipsychotics improve this tonic hyperactivation of thalamocortical glutamatergic transmission [38,41,43,44,45,46,47,60,67,68,69,114,129,130,159,166,167,168].

The local administration of several antipsychotics into the mediodorsal thalamic nucleus (MDTN) has been demonstrated to inhibit MK801-induced tonic activation of glutamatergic transmission in the thalamocortical pathway by suppressing glutamatergic neuronal activity in the MDTN [159,164,168]. In addition, local administration of clozapine into the mPFC suppresses MK801-induced hyper-glutamatergic transmission [42]. Studies have shown that the suppressive actions of clozapine on the tonic activation of thalamocortical glutamatergic transmission are predominant in the cortex rather than the thalamus, which is a distinguishing feature compared with other atypical antipsychotics [38,41,42,43].

## 5. Conclusions

Based on the accumulated recent clinical and preclinical findings, the present review introduced the possibility that L-BAIBA, a novel protective myokine in the peripheral organs, plays important roles in the mechanisms of the clinical actions of clozapine regarding its efficacy in treating TRS and its adverse reactions, such as weight gain and metabolic complications. In peripheral organs, L-BAIBA improves insulin-resistant diabetes by activating AMPK, and activated AMPK in the hypothalamus leads to weight gain. The suppression of IP3 synthesis through the inhibition of the H1 and 5-HT2A receptors has been considered a major mechanism of weight gain and metabolic complications associated with atypical antipsychotics. Low-risk atypical antipsychotics for weight gain (brexpiprazole and lurasidone) and clozapine decrease IP3 synthesis, leading to increasing intracellular AMP levels, whereas the effects on AMPK activity are different between low-risk antipsychotics for weight gain and clozapine. Both brexpiprazole and lurasidone do not activate AMPK signalling, but clozapine activates AMPK signalling in the hypothalamus. Therefore, L-BAIBA contributes to the pathophysiology of clozapine-induced metabolic complications via independent IP3 pathways. Persistent tonic hyperactivations of thalamocortical glutamatergic transmission produce sensorimotor deficits, which are considered a major component of cognitive impairment in TRS. Increasing L-BAIBA signalling in both the thalamus and prefrontal cortex attenuates the persistent tonic hyperactivations of thalamocortical glutamatergic transmission induced by NMDA-R disturbance via the activation of the GABA-A, GABA-B, glycine, and III-mGlu receptors. These recent preclinical findings suggest that the stimulatory effects of clozapine on L-BAIBA are, at least partially, involved in the mechanisms of clozapine’s clinical actions, such as its efficacy in TRS and metabolic complications.

## Data Availability

The data that support the findings of this study are available from the corresponding author upon reasonable request. Some data may not be made available because of ethical restrictions.
